# On the Effect of Microwave Energy on Lipase-Catalyzed Polycondensation Reactions

**DOI:** 10.3390/molecules21091245

**Published:** 2016-09-19

**Authors:** Alessandro Pellis, Georg M. Guebitz, Thomas J. Farmer

**Affiliations:** 1 Institute for Environmental Biotechnology, University of Natural Resources and Life Sciences, Konrad Lorenz Strasse 20, 3430 Tulln an der Donau, Austria; alessandro.pellis@gmail.com (A.P.); guebitz@boku.ac.at (G.M.G.); 2 Green Chemistry Centre of Excellence, Department of Chemistry, University of York, Heslington, York YO10 5DD, UK; 3 Austrian Centre of Industrial Biotechnology GmbH, Konrad Lorenz Strasse 20, 3430 Tulln an der Donau, Austria

**Keywords:** *Candida antarctica* lipase B, microwave energy, polycondensation reactions, enzymatic synthesis, bio-based monomers, bio-based polymers, platform molecules

## Abstract

Microwave energy (MWe) is, nowadays, widely used as a clean synthesis tool to improve several chemical reactions, such as drug molecule synthesis, carbohydrate conversion and biomass pyrolysis. On the other hand, its exploitation in enzymatic reactions has only been fleetingly investigated and, hence, further study of MWe is required to reach a precise understanding of its potential in this field. Starting from the authors’ experience in clean synthesis and biocatalyzed reactions, this study sheds light on the possibility of using MWe for enhancing enzyme-catalyzed polycondensation reactions and pre-polymer formation. Several systems and set ups were investigated involving bulk and organic media (solution phase) reactions, different enzymatic preparations and various starting bio-based monomers. Results show that MWe enables the biocatalyzed synthesis of polyesters and pre-polymers in a similar way to that reported using conventional heating with an oil bath, but in a few cases, notably bulk phase polycondensations under intense microwave irradiation, MWe leads to a rapid enzyme deactivation.

## 1. Introduction

The drive towards a Circular Economy will look to chemistry and biology to provide the basis of innovative products, made from renewable feedstocks and designed to be reused, recycled, or the feedstock renewed through controlled or natural processes [1]. In particular, the polymer industry is under pressure to mitigate the environmental cost of using petroleum-based plastics, seeking both to source monomers from biomass and to carry out the synthesis of the polymers in a more benign fashion. Biotechnology is already contributing significantly to the gradual replacement of petroleum-based chemicals, thus bringing closure of the carbon circle [2]. The need now is for biotechnology to have a greater impact in developing benign polymerization processes. Several reports on enzymatic synthesis of bio-based polyesters have been presented in the last decade, leading to the production of aliphatic [3,4], aromatic [5,6] and functional [7,8] products, achieving materials otherwise unlikely obtainable via conventional chemical routes [9,10].

The most studied and well-characterized enzyme able to catalyze such reactions is *Candida antarctica* lipase B (CaLB), a thermostable biocatalyst mostly used in its immobilized form [5,6,8]. Despite the biocatalyst’s wide stability towards temperature (up to 90 °C) and organic solvents, several further attempts to improve its performance have also been investigated, involving mainly the immobilization formulation [11,12], ultrasound [13,14] and microwave energy [15,16]. Indeed, microwave energy (MWe) offers several benefits as a greener way of heating chemical reactions [17,18,19,20]:

- Microwave energy can be introduced remotely without the need for direct contact between the energy source and the chemicals, thus avoiding hot spots and improving product selectivity
- Energy input starts and stops immediately when power is turned on or off, thus when hazardous exotherms are encountered the MW irradiation can be instantly removed
- Heating rates are high as long as one of the components of the reaction can couple strongly with the microwaves
- Energy consumption is generally considerably lower for microwave assisted reactions as the reaction media or reagents are heated directly without prior need of heating the reaction vessel and surrounding equipment

**Figure 1:**
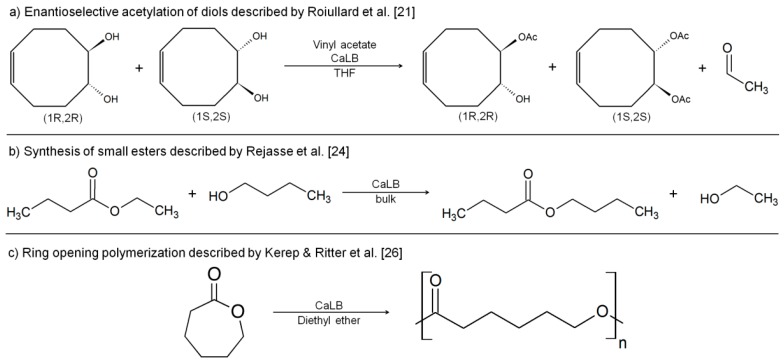
MWe-assisted CaLB-catalyzed reactions, already reported in the literature.

Most of the previous literature on CaLB and the use of MWe focuses on the processing of small molecules, such as kinetic resolutions of (*Z*)-Cyclooct-5-ene-1,2-diol and (*Z*)-2-Acetoxycyclooct-4-enyl acetate (Figure 1a) [21], biodiesel production [22], synthesis of small esters (Figure 1b) [23,24,25,26] and the characterization of the biocatalyst selectivities [27]. Despite extensive prior research into the use of biocatalysis for ester formation or reactions, only limited attempts have been made for the biocatalyzed synthesis of polyesters.

Previous literature on MWe-assisted enzymatic synthesis has focused on ring opening polymerizations (ROP) of ε-caprolactone (ε-CL). Kerep and Ritter investigated the ROP of ε-CL in various organic solvents (used at their respective boiling points), discovering that a reaction yield improvement is obtained for MWe when diethyl ether was used as solvent, if compared to the classical reaction in an oil bath (Figure 1c) [28]. Interestingly, this trend was observed only for this slightly polar and low boiling point ether solvent but not for benzene or toluene, solvents where this enzymatic reaction is traditionally conducted under conventional heating. In a follow-up paper the authors describe how copolymers of polycaprolactone (PCL) and polystyrene can be obtained using MWe and 2-mercaptoethanol as ROP initiator [29]. Matos et al. reported the successful use of MWe heating for the ROP of ε-CL in solvent-free conditions (90 °C, 240 min, 50 W). In this work an experimental design showed a four-fold increase of the M_n_ of PCL when MWe was used compared to conventional heating via an oil bath [30].

Based on these previous reports that certify how CaLB is active in enzymatic ring-opening polymerization reactions under MWe heating, the present work instead focuses on the synthesis of bio-based polyesters via trans-esterification reactions. Such reaction systems involving a biocatalyzed reaction between a diester and a diol under MWe were never previously investigated. Moreover, an investigation into biocatalyzed MWe trans-esterification is of current interest since such reactions, but under conventional heating, are widely used for the production of bio-based polyesters and generally seen as greener option compared to reactions using diacyl chlorides [31,32]. Additionally, ROP requires the synthesis of monomers containing adequate ring-strain for the polymerization to be carried out, this reducing significantly the total number of possible monomers that can be used, whilst also relying on low atom-economic synthetic procedures such as Baeyer-Villiger oxidations. Trans-esterification polymerization reactions are instead applicable to a far wider choice of monomers (diacids/esters, diols, polyols and hydroxyacids) many of which are readily available from biomass [33,34]. Therefore, demonstration of how trans-esterification can be coupled with enzymes under MWe heating for the formation of sustainable polyesters would represent a major advancement in the application of green technologies and be highly relevant to the utilization of biomass derived chemicals. A limitation previously observed for enzyme catalyzed transesterification polymerizations is the issue of achieving large chain lengths, especially under low temperatures (<100 °C) and at ambient pressure. As a result a more common approach when producing polyesters catalyzed by enzymes is to carry out an initial oligomerization, forming low molecular weight (<1000 Da) pre-polymers [2,7,35]. These pre-polymers can be recovered more easily from the enzyme containing media due to a lower viscosity compared to higher molecular mass chains. If desired these pre-polymers can further be upgraded or reacted at reduced pressures, higher temperatures or through the addition of a small amount of a more reactive monomer acting as chain extender or cross-linkers [36,37,38].

## 2. Materials and Methods

### 2.1. Materials and Enzymes

All chemicals and solvents were purchased from Sigma-Aldrich and used without further purification unless otherwise specified. Novozym^®^ 435 is a commercial formulation (Sigma-Aldrich Company Ltd., Dorset, UK) of lipase B from *Candida antarctica* (CaLB), adsorbed on a macroporous methacrylic resin. A covalently immobilized CaLB preparation on EC/EP-M beads (Resindion S.r.l, Milan, Italy) was also used [3]. The immobilized enzymes were dried over phosphorous pentoxide for 48 h under reduced pressure prior to use, as previously described in earlier examples of its use [6,35].

### 2.2. Polycondensation Reactions

#### 2.2.1. Solvent-Free Reactions

Amounts of 0.01 mol dimethyl ester (A) and 0.01 mol diol (B) were weighed into a 10-mL reaction vessel and stirred at 50 °C until a homogeneous solution was obtained. The biocatalyst (10% w·w^−1^ relative to the total amount of monomers) was then added to the reaction mixture. The reaction tube (10-mL) was placed into a CEM Microwave Discover™ synthesizer at a preset temperature, time, and MW power settings. The contents of the reaction vessel were stirred by a magnetic stir bar (high speed). Temperature, pressure, and power were constantly monitored using the software provided by the MW manufacturer. For some reactions, the Power Max option was used; a flow of compressed air was applied in order to maintain the preset reaction temperature and allow for a greater intensity of MW irradiation to be applied. Samples were withdrawn at several time intervals and the crude reaction mixture was analyzed via ^1^H-NMR spectroscopy and GPC without prior purification steps.

The same reaction set up (amounts, reaction tube etc.) was used in a classical oil bath reaction system for a direct comparison.

#### 2.2.2. Reactions in Organic Media

Amounts of 0.004 mol dimethyl ester (A) and 0.004 mol diol (B) were weighed into a 30-mL reaction vessel. 10-mL of diethyl ether was then added and the mixture stirred at 30 °C until a homogeneous clear solution was obtained. The biocatalyst (10% w·w^−1^ relative to the total amount of monomers) was then added to the reaction mixture. The reaction tube was placed into a CEM Microwave Discover™ synthesizer at a preset temperature, time, and MW power setting. The contents of the reaction vessel were stirred by a magnetic stir bar. Temperature, pressure, and power were constantly monitored using the software provided by the MW manufacturer. For some reactions, the Power Max option was applied (described in Section 2.2.1). Samples were withdrawn at several time intervals and the crude reaction mixture was analyzed via ^1^H-NMR spectroscopy and GPC without prior purification steps.

The same reaction set up was used in a classical oil bath reaction system for comparison.

### 2.3. ^1^H-NMR Spectroscopy Analysis

^1^H-NMR spectroscopy measurements were performed on a Jeol 400 spectrometer (resonance frequency of 400.13 MHz for ^1^H). CDCl_3_ was used as NMR spectroscopy solvent unless otherwise specified (see Figure S1 for the assigned ^1^H-NMR spectra of a typical reaction).

### 2.4. Gel Permeation Chromatography (GPC)

GPC Samples were dissolved in THF (250 ppm BHT as inhibitor) and filtered through 0.2 μm PTFE filters using a syringe. In case of liquid samples, the starting solvent was removed under reduced pressure using a rotary evaporator. Gel permeation chromatography was carried out at 30 °C on an Agilent Technologies HPLC 1260 Infinity System (Agilent Technologies UK Ltd., Craven Arms, UK) connected to a 6.0 mm ID × 40 mm L HHR-H, 5 μm Guard column (17369) and a 7.8 mm ID × 300 mm L GMHHR-N, 5 μm TSK gel liquid chromatography column (18055, Tosoh Bioscience, Tessenderlo, Belgium) using THF (250 ppm BHT as inhibitor) as eluent (flow rate 1 mL·min^−1^). An Agilent Technologies G1362A refractive index detector was employed for detection. The molecular weights of the polymers were calculated using linear polystyrene calibration standards (250–70,000 Da). The injection volume was 40 μL.

## 3. Results and Discussion

### 3.1. Solvent-Free Reactions

**Figure 2:**
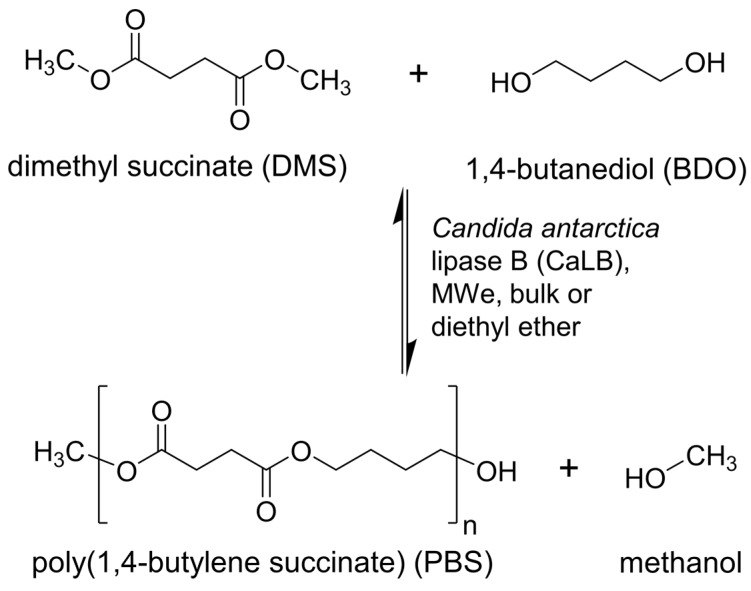
The investigated polycondensation reaction involving the transesterification of dimethyl succinate (DMS) with 1,4-butanediol (BDO) catalyzed by an immobilized preparation of *Candida antarctica* lipase B (CaLB).

**Figure 3:**
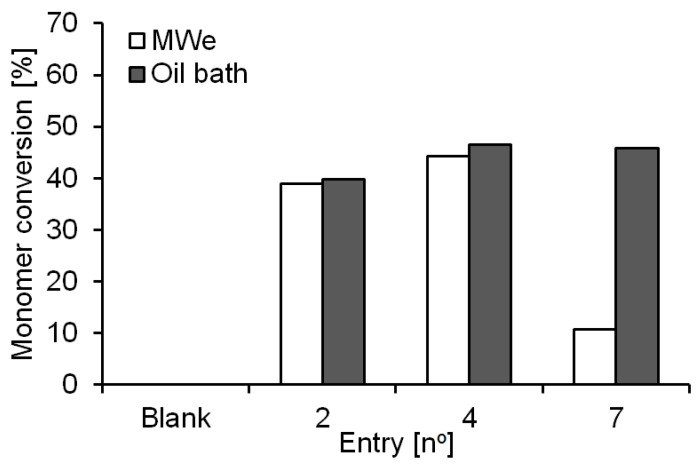
Monomers conversion overview from the bulk system reactions catalyzed by Novozym^®^ 435 at 50 °C and power of 200 W after 4 h. Conversions were calculated from ^1^H-NMR analysis of the crude reaction products. 2: closed vessel; 4: open vessel; 7: Power Max function.

In a first instance we investigated the effect of MWe on solvent-free (bulk) reactions (see Figure 2 for the reaction scheme), as previously reported by Rejasse et al. for the synthesis of butyl butyrate using both free and immobilized CaLB [23,24]. The first polycondensation attempts were performed using a closed reaction system irradiating the reaction mixtures with MWe intensely just in the beginning and afterwards applying only a few Watts (2–5 W) of MWe energy in order to maintain the desired temperature (Table 1, entries 1–3). From these initial experiments MWe heating was found to give comparable results to traditional oil bath heating, with monomers conversion (Figure 3) and weight average molecular weights (M_w_) being almost identical (Table 1, entries 1–3). Additionally, changing the maximum power (set max) of the reaction did not lead to any appreciable change to the conversion rates (See Table S1 in ESI for details on conversions and molecular weights). It should be noted the at the maximum power setting for these fix temperature runs only alters the maximum wattage that the machine can apply to reach the desired temperature, but, in this instance, the overall power supplied for the reaction was so low that there is negligible difference between a set max of 100 W or 200 W.

From prior research into conventionally heated enzyme-catalyzed transesterifications it is known that CaLB shows better activity when the reaction byproduct (in this case methanol) is removed [6,8], thus, an open vessel systems was investigated. When the exact reaction conditions (described above for entries 1–3) were used, no MWe effects on the reactions were observed (Table 1, entries 4–6). Using the Power Max option of the CEM Microwave Discover™, as previously described [30], instead resulted in lower monomer conversion rates and similar M_w_ values (Table 1, entry 7). Increasing the operating temperature from 50 °C to 70 °C did not lead to any improvements in conversion or M_w_ (Table 1, entry 4 versus 6 and entry 7 versus 8). However, it was observed that both reactions conducted using the Power Max function (i.e., increased MWe irradiation intensity, entries 7 and 8) resulted in a final yellowish reaction mixture while the reactions conducted in the oil bath resulted in clear colorless solutions. Since microwaving of the monomers alone did not show a difference in the conversion or any color change we suppose that such phenomena is derived from the enzymatic preparation instead. Similar observations of a color change were made when a covalently immobilized CaLB preparation [3] was used. An overview of the monomer conversions of the bulk reaction after 4 h is presented in Figure 3 where it is possible to notice how for entries 2 and 4 (reaction with closed and open vessel respectively) no conversion differences between MWe and conventional heated were detected. However, for entry 7 (Power Max function) a 75% reduction of the monomers conversion was noticed for MWe heating.

The results obtained from the solvent-free polymerizations in bulk systems are in line with what was previously reported by Rejasse et al. on the CaLB-catalyzed small molecule synthesis of butyl butyrate [23,24]. In fact, no MWe improvement on the monomer conversion rate was observed in comparison with the oil bath reaction system. However, a detrimental effect on the biocatalyst was instead noticed when the Power Max mode was applied. The basis of such negative effects are currently under further investigation, but would seemingly be linked to denaturing of the enzyme when put under higher levels of microwave irradiation. This observation is discussed in greater detail below in comparison to equivalent reactions carried out in solution phase (organic media).

### 3.2. Reactions in Organic Media

There are several benefits to carrying out polymerizations in solution phase, such as reduced viscosity, improved miscibility/mixing of monomers and increased rates of diffusion in and out of the catalysts porous structure. Following previous reports on the ROP of ε-CL [28,29], we selected diethyl ether as reaction solvent since previous trials using benzene and toluene did not lead to any reaction yield improvement [28]. Reactions conducted at 30 °C (temperature below the boiling point of the solvent) and at 38 °C (solvent’s boiling temperature as previously determined by Kerep and Ritter [28]) did not show any significant difference for monomer conversion when compared with the conventional oil bath system (Table 2, entries 9–11). Interestingly, using the Power Max function in organic media did not lead to any remarkable inhibition of the biocatalyst preparation, with conversions after 4 h of reaction of 47% and 48% for the MWe and the oil bath reaction, respectively (Table 2, entries 12 and 13, further details in Table S2).

Contrary to what was previously reported by Kerep and Ritter regarding the ROP of ε-CL [28,29], we did not observe any effect, either positive or negative, for using diethyl ether as reaction solvent in comparison to bulk polycondensation/transesterification reactions between dimethyl esters and 1,4-butanediol. However, it should be noted that, for our reaction set up a condenser was used in order to avoid solvent loss during the polymerization, this therefore possibly limiting the removal of the volatile methanol co-product. This would not have affected our direct comparison of MWe and conventional heating as the exact same reaction set up was used for both (i.e., condenser fitted to both), but may have caused the difference in observed affects compared to Kerep and Ritter’s previous study, where there was no mention of a condenser. An argument against this hypothesis is that the ROP studied by Kerep and Ritter does not generate a reaction co-product and therefore loss of the solvent should not pull the reaction to higher conversions, unless the boiling point of the whole system increased as a result of solvent loss. The discussion represents and excellent example of why extreme care must be taken when comparing conventional heating with MWe heating as every effort must be made to ensure that the reaction set-ups are identical, including the use of condensers and monitoring solvent loss.

**Figure 4:**
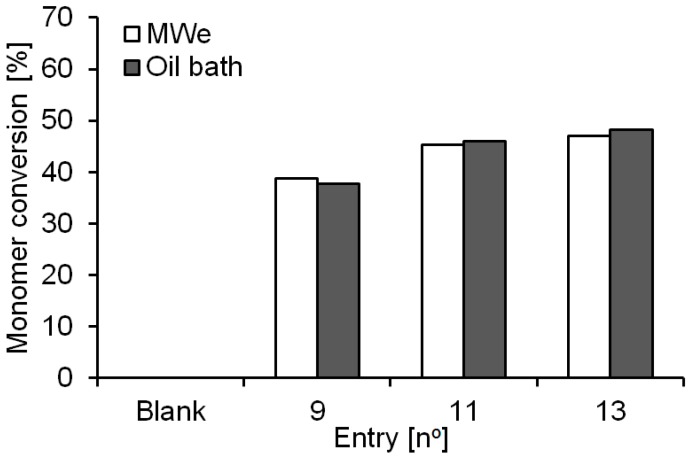
Monomers conversion overview from the reactions conducted in organic solvents catalyzed by Novozym^®^ 435 at 30 °C (entry 9) and 38 °C (entries 11 and 13). Conversions were calculated from ^1^H-NMR spectra of the crude reaction mixture.

An overview of our monomer conversions of the polycondensations conducted in diethyl ether solvent after 4 h of reaction is presented in Figure 4. No appreciable difference was observed between MWe and conventional oil bath heating, even when conditions are altered such as non-boiling (entry 9) and boiling solvent (entry 11) or the use of Power Max (entry 13, compared to entry 11). Under Power Max conditions an increased intensity of microwave irradiation is applied by continually cooling the reaction vessel via a flow of compressed air (see Table S2 in ESI for details on conversions and molecular weights).

**Figure 5:**
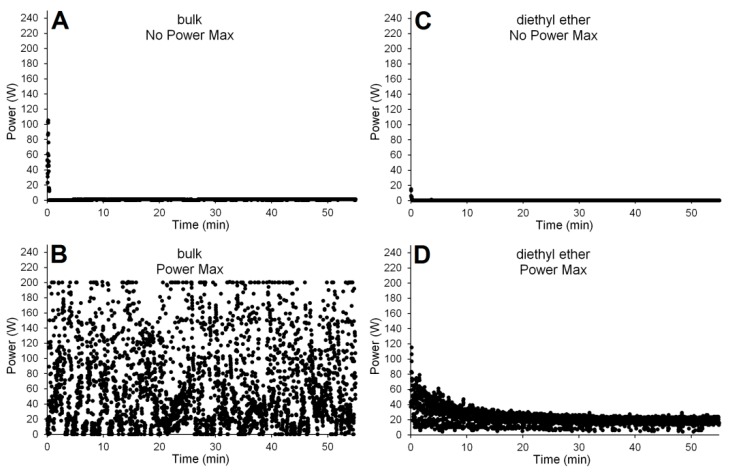
Microwave energy erogation plots over time for (**A**) bulk/no Power Max; (**B**) bulk/Power Max; (**C**) diethyl ether/no Power Max and (**D**) diethyl ether/Power Max reactions between DMS and BDO. For the Power Max function, the maximum reachable power was set to 200 W, temperature was kept constant via a continuous compressed air flow.

This observation of no negative effects of Power Max differs to that previously observed for the bulk reaction, where Power Max significantly reduced monomer conversion (−70% difference). Figure 5 shows the scatter plots of the power input versus time for four key reactions. As can be seen for both the bulk and diethyl ether runs with no Power Max (Figure 5A,C, respectively) the power profiles show that very briefly at the start of the reaction the input energy is moderate (<100 W), but remains very low (<5 W) after the initial minute of the reaction. As a result, the average power for the duration of these two reactions was very low, just 1.01 and 0.02 W per second (where 1 W per second is equivalent to 1 J), respectively (entries 4a and 11a, Table 3), with this power required only to maintain their reaction temperatures (30 °C and 50 °C, respectively). In comparison, the Power Max reaction, using the diethyl ether solvent, had an average power input of 21.58 W, this being considerably more than the 0.02 W for the aforementioned equivalent non-Power Max reaction. However, the Power Max diethyl ether reaction (entry 13a, Table 3) gave no positive or negative effect in conversion or molecular weight compared to the comparative non-Power Max (entry 11a), showing that irradiation at this level was not problematic for the enzyme. As described above, comparing the bulk non-Power Max and Power Max a difference in monomer conversion was observed, with Power Max proving detrimental to conversion (entry 7, Figure 3). The power profile of the bulk Power Max (Figure 5B) shows that often very high intensity irradiation was applied, and the average power of this run was 64.49 W, more than 60 times higher than the equivalent bulk non-Power Max (Figure 5A). The bulk Power Max profile clearly shows that the power fluctuates greatly between low and very high intensity irradiation, and it is likely that this caused denaturing of the enzyme and subsequent reduced monomer conversion (entry 4a versus 7a, Table 3). This hypothesis is further supported by comparing the Power Max profiles of the bulk (Figure 5B and entry 7a) and the solution phase (Figure 5D and entry 13a) reactions where the solution phase is much more controlled with no obvious power spikes. This is reasoned as the solvent acts as a heat sink, cushioning the effects of the irradiation, thus reducing enzyme denaturing. On this evidence it would suggest that enzyme catalyzed bulk polytransesterification reactions are sensitive to elevated levels of microwave irradiation and that modes, such as Power Max, should be avoided.

## 4. Conclusions

Over the years, several reports of MWe applied to biocatalyzed reactions have been published but, to the best of our knowledge, only the ROP of ε-CL has been carried out when investigating enzymatic polymer synthesis. In this work we investigated the effects of MWe for the first time on the polycondensation of dimethyl esters with 1,4-butanediol using the commercially available CaLB as the biocatalyst. Using the reported reaction conditions MWe does not have any notable effect, positive or negative, on the biocatalyzed synthesis of polyesters in polycondensations, giving monomer conversion rates and Mw similar to the reactions conducted in a conventional oil bath. For these comparisons, this study was meticulous in ensuring that the reaction set-up between MWe and conventional heating were as similar as possible. Only when using the Power Max function for the bulk solvent-free polymerization system was a difference noticed between MWe and conventional, with a reduced monomer conversion observed for MWe. Intriguingly, when comparing Power Max solution (diethyl ether) phase polymerization versus conventional heating no difference was observed, indicating that it was only in bulk polymerizations under high microwave irradiation that negative effects on the enzyme are observed. Bulk Power Max reactions were found to suffer from power spikes when using our chosen reaction set-up and we attribute this to the observed denaturing of the enzyme. This effect is still under further investigation since it is well known that in certain conditions MWe could cause alterations of the local configuration of the biocatalyst, leading to its rapid deactivation. Apart from issues with bulk polymerization, this study has shown that MWe can be used as a suitable replacement for conventional heating for the enzyme catalyzed polycondensation of bio-derived polyesters and diols with minimal impact on monomer conversion and eventual polymer chain length.

## Figures and Tables

**Table 1:** Solvent-free reactions catalyzed by Novozym 435^®^ after 4 h ^@^ of reaction.

Entry (No.)	Diester (A)	Diol (B)	Power (W)		T (°C)	Vessel	Difference vs. Oil Bath	
			Set Max	Power Max		Open/Close	Conversion (%) *	M_w_ (Da) ^λ^
1	DMA	BDO	200	-	50	Close	=	=
2	DMS	BDO	200	-	50	Close	=	=
3	DMS	BDO	100	-	50	Close	=	=
4	DMS	BDO	200	-	50	Open	=	−173
5	DMA	BDO	100	-	50	Open	=	−125
6	DMS	BDO	200	-	70	Open	=	=
7	DMS	BDO	200	+	50	Open	−75	−220
8	DMS	BDO	200	+	70	Open	−60	−114

* Calculated via ^1^H-NMR; difference vs. oil bath calculated as %conversion for MWe minus %conversion for conventional oil bath heating; ^λ^ Calculated via GPC; difference vs. oil bath calculated as M_w_ for MWe minus M_w_ for oil bath heating. M_w_ were considered equal when the difference was <100 Da; ^@^ Initial time course experiments (1 h to 6 h, see Supplementary Materials Figures S2–S4) were used to determine that 4 h was a suitable point of reference with adequate yields for conditions tested. Abbreviations: DMA: dimethyl adipate; DMS: dimethyl succinate; BDO: 1,4-butanediol. Further comprehensive experimental details such as conversions and M_w_ for each experiment can be found in the electronic Supplementary Materials (Table S1).

**Table 2:** Reactions in organic media catalyzed by Novozym 435^®^ after 4 h of reaction.

Entry (No.)	Diester (A)	Diol (B)	Power (W)		T (°C)	Difference vs. Oil Bath	
			Set Max	Power Max		Conversion (%) *	M_w_ (Da) ^λ^
9	DMS	BDO	200	-	30	=	=
10	DMA	BDO	200	-	30	=	=
11	DMS	BDO	200	-	38	=	=
12	DMS	BDO	100	+	38	=	=
13	DMS	BDO	200	+	38	=	=

* Calculated via ^1^H-NMR; difference vs. oil bath calculated as %conversion for MWe minus %conversion for oil bath heating; ^λ^ Calculated via GPC; difference vs. oil bath calculated as M_w_ for MWe minus M_w_ for oil bath heating. M_w_ were considered equal when the difference was <100 Da. Abbreviations: DMA: dimethyl adipate; DMS: dimethyl succinate; BDO: 1,4-butanediol. Further comprehensive experimental details such as conversions and M_w_ for each experiment can be found in the Supplementary Materials (Table S2).

**Table 3:** Comparison of average power over 60 min for bulk and organic media reactions with or without Power Max of DMS with BDO.

Entry (No.)	Reaction Type	Power Max	T (°C)	Time (min)	Av. Power (W/s)	Conversion (%) *
11a	Diethyl ether	No	38	60	0.02	45
13a	Diethyl ether	Yes	38	60	21.58	47
4a	Bulk	No	50	60	1.01	47
7a	Bulk	Yes	50	60	64.49	11

* Calculated via ^1^H-NMR; difference vs. oil bath calculated as %conversion for MWe minus %conversion for conventional oil bath heating; Calculated via ^1^H-NMR, a = only MWe heated reactions included.

## Supplementary Materials

Supplementary materials can be accessed at: http://www.mdpi.com/1420-3049/21/9/1245/s1.

## Access Statement

All data used in the preparation of this manuscript for the sections funded by the EPSRC grant EP/L017393/1 is contained within this document, the ESI or available via a depository (doi:10.15124/49496b1b-9109-46bb-8175-6d8455ffc964).

## Abbreviations

The following abbreviations are used in this manuscript:

MWe	microwave energy
CaLB	*Candida antarctica* lipase B
ROP	ring opening polymerization
ε-CL	ε-caprolactone
DMA	dimethyl adipate
DMS	dimethyl succinate
BDO	1,4-butanediol
