# Supplementary material for: On the Effect of Microwave Energy on Lipase-Catalyzed Polycondensation Reactions
Source: Molecules. 2016 Sep 19;21(9):1245. doi: 10.3390/molecules21091245 (PMC6274407; doi:10.3390/molecules21091245)
Supplement: Supplementary file 1 [file molecules-21-01245-s001.pdf]

Supplementary Materials: On the Effect of Microwave energy on Lipase-Catalyzed Polycondensation Reactions

Alessandro Pellis, Georg M. Guebitz and Thomas J. Farmer

Table S1. Solvent-free reactions catalyzed by Novozym 435® after 4 h of reaction.

Entry (n°)	Diester (A)	Diol (B)	Heating		T (°C)	Vessel	Conversion (%) *	M_w (Da) ^	M_n (Da) ^	PDI ^
			MW	Oil Bath		Open/Close				
Blank	DMA	BDO	+		50	Open	-	-	-	-
Blank	DMA	BDO		+	50	Close	-	-	-	-
2	DMA	BDO	+		50	Close	39	509	483	1.054
2	DMA	BDO		+	50	Close	40	515	488	1.055
4	DMS	BDO	+		50	Open	44	546	456	1.197
4	DMS	BDO		+	50	Open	47	599	528	1.134
7	DMS	BDO	+		50	Open	11	391	335	1.167
7	DMS	BDO		+	50	Open	46	611	543	1.125

* Calculated via ^{1}H-NMR spectra; ^ Calculated via GPC. Abbreviations: DMA: dimethyl adipate; DMS: dimethyl succinate; BDO: 1,4-butanediol.

Table S2. Reactions in organic media catalyzed by Novozym 435® after 4 h of reaction.

Entry (n°)	Diester (A)	Diol (B)	Heating		T (°C)	Conversion (%) *	M_w (Da) ^	M_n (Da) ^	PDI ^
			MW	Oil Bath					
Blank	DMS	BDO	+		38	-	-		
Blank	DMS	BDO		+	38	-	-		
9	DMS	BDO	+		30	39	373	330	1.130
9	DMS	BDO		+	30	38	303	287	1.056
11	DMS	BDO	+		38	45	480	399	1.203
11	DMS	BDO		+	38	46	479	391	1.225
13	DMS	BDO	+		38	47	553	514	1.076
13	DMS	BDO		+	38	48	530	505	1.050

* Calculated via ^{1}H-NMR spectra; ^ Calculated via GPC. Abbreviations: DMA: dimethyl adipate; DMS: dimethyl succinate; BDO: 1,4-butanediol.

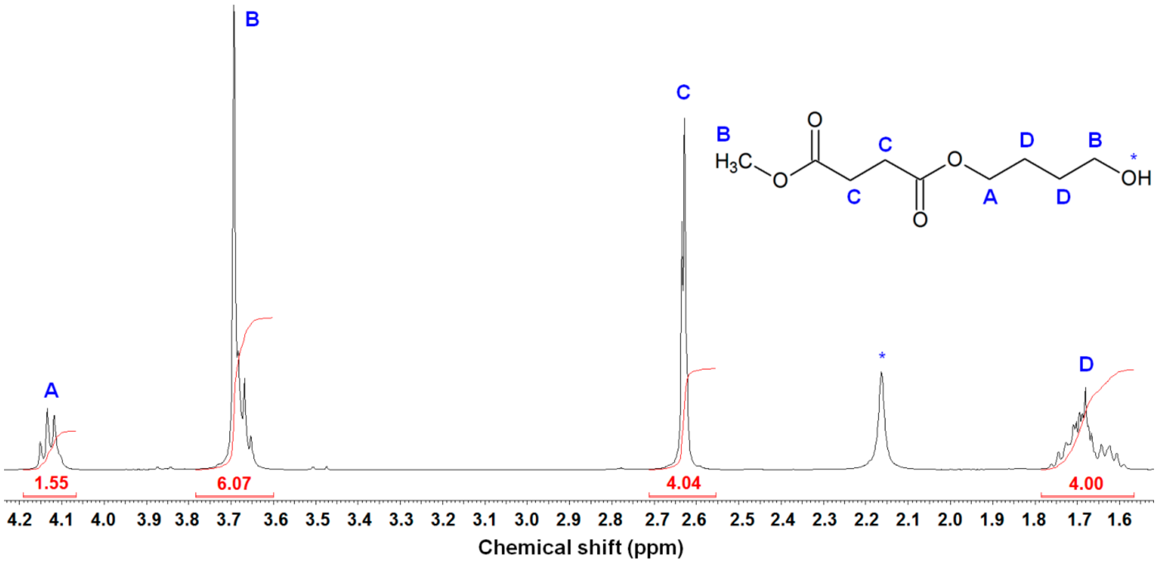

Figure S1. ^{1}H-NMR spectrum of the polycondensation products of DMS with BDO catalyzed by 10% w·w Novozym® 435 at 4 h. Entry 2 Table 1 and Table S1.

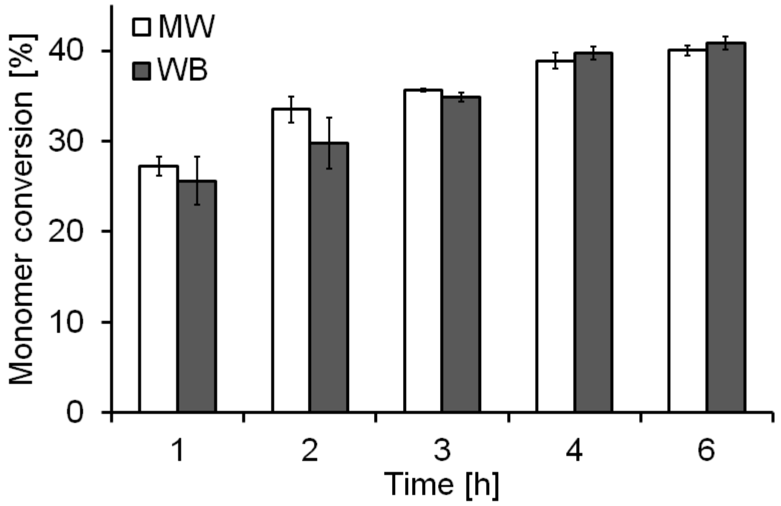

Figure S2. Time-course monitoring of the monomers conversion via ^{1}H-NMR spectra of the bulk reaction performed in a closed vessel at 50 °C without using the Power Max function.

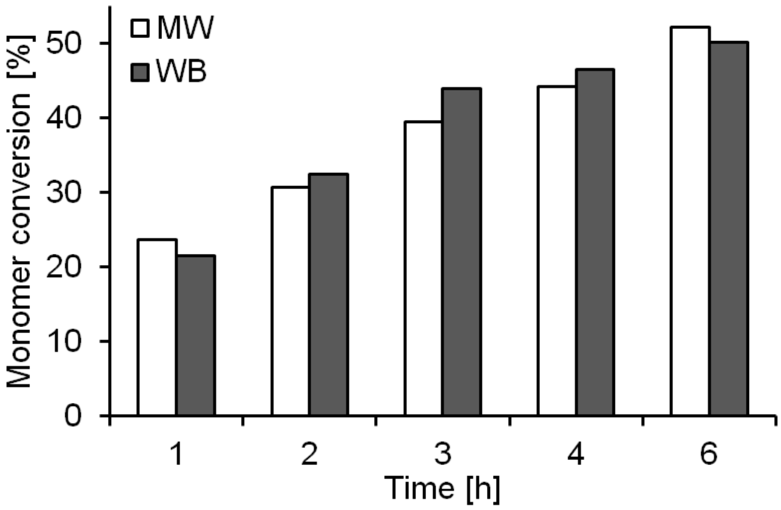

Figure S3. Time-course monitoring of the monomers conversion via ^{1}H-NMR spectra of the bulk reaction performed in an open vessel at 50 °C without using the Power Max function.

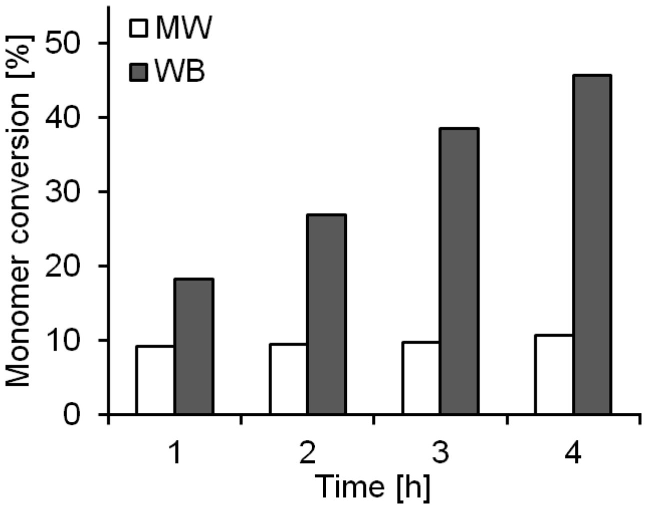

Figure S4. Time-course monitoring of the monomers conversion via ^{1}H-NMR spectra of the bulk reaction performed in an open vessel at 50 °C using the Power Max function.
